# Study of Wound Healing Dynamics by Single Pseudo-Particle Tracking in Phase Contrast Images Acquired in Time-Lapse

**DOI:** 10.3390/e23030284

**Published:** 2021-02-26

**Authors:** Riccardo Scheda, Silvia Vitali, Enrico Giampieri, Gianni Pagnini, Isabella Zironi

**Affiliations:** 1 DIFA-Physics and Astronomy Department, University of Bologna, Viale C. Berti Pichat 6/2, 40127 Bologna, Italy; riccardo.scheda@studio.unibo.it (R.S.); isabella.zironi@unibo.it (I.Z.); 2 BCAM-Basque Center for Applied Mathematics, Alameda de Mazarredo 14, 48009 Bilbao, Spain; gpagnini@bcamath.org; 3 eDIMESlab, Department of Experimental, Diagnostic and Specialty Medicine, University of Bologna, Via Irnerio 49, 40126 Bologna, Italy; enrico.giampieri@unibo.it; 4 Ikerbasque-Basque Foundation for Science, Plaza Euskadi 5, 48009 Bilbao, Spain

**Keywords:** wound healing dynamics, single pseudo-particle tracking, phase contrast image segmentation

## Abstract

Cellular contacts modify the way cells migrate in a cohesive group with respect to a free single cell. The resulting motion is persistent and correlated, with cells’ velocities self-aligning in time. The presence of a dense agglomerate of cells makes the application of single particle tracking techniques to define cells dynamics difficult, especially in the case of phase contrast images. Here, we propose an original pipeline for the analysis of phase contrast images of the wound healing scratch assay acquired in time-lapse, with the aim of extracting single particle trajectories describing the dynamics of the wound closure. In such an approach, the membrane of the cells at the border of the wound is taken as a unicum, i.e., the wound edge, and the dynamics is described by the stochastic motion of an ensemble of points on such a membrane, i.e., pseudo-particles. For each single frame, the pipeline of analysis includes: first, a texture classification for separating the background from the cells and for identifying the wound edge; second, the computation of the coordinates of the ensemble of pseudo-particles, chosen to be uniformly distributed along the length of the wound edge. We show the results of this method applied to a glioma cell line (T98G) performing a wound healing scratch assay without external stimuli. We discuss the efficiency of the method to assess cell motility and possible applications to other experimental layouts, such as single cell motion. The pipeline is developed in the Python language and is available upon request.

## 1. Introduction

In this paper, we propose a pipeline for the segmentation and analysis of phase contrast images acquired in time-lapse in the wound healing scratch assay, to overcome some limitations of standard approaches due to the change in shape and density of the cells during migration.

Cellular migration is a fundamental process for animal’s physiology during both the period of development and that of maturity. Cells migrate to shape organs and tissues and, in the case of damage, regenerate them. Furthermore, motility is a primary skill in cancer metastatic processes and in the immune responses [1,2]. The capability to migrate is a highly regulated process in which cells respond to external and internal mechanical, electrical, and chemical stimuli by complex physiological processes that promote, enhance, or suppress cell motility [3,4]. Cells can be induced to move in a particular direction by positive and negative guidance signals, while in the absence of external guidance, cells move randomly [5,6].

In cutaneous wound healing, which is a complex cellular and biochemical process necessary to restore structurally damaged tissue, skin cells migrate from the wound edges towards the empty space to restore skin integrity. In this case, the cohesive group of cells organized in a layer modifies the classical characteristics of single cell migration, and the presence of the wound induces peculiar migration behaviors. In fact, while a certain freedom of movement is maintained inside the tissue, the cells along the edge of the wound (front) move preferentially toward the gap. Such a process involves dynamical interactions between both the contacting cells (which are absent in single cell migration) and the extracellular matrix. These interactions regulate motility enhancement or suppression [7,8].

The wound healing scratch assay is a widespread experimental tool applied to study the collective migration of cells cultured in vitro. Standard protocols provide that a highly confluent monolayer of cells is scratched by a fine pipette tip to create a gap, which is then allowed to heal. As a protocol of analysis, the area of the scratch is measured as a function of time to determine the speed of the closure. This method is meant to simulate a natural wound, and the procedure is simple and easy to set up, but it is difficult to analyze and produce precise and reproducible results [9].

The mathematical continuum models that focus on the collective properties of cells can explain the requirements for the onset of movement and some typical characteristics of cell motility, but are usually limited to small space-time scales. Therefore, they provide little information on how the integration of the lamellipodium protrusion, the retraction of the posterior part, and the transduction of force on the extracellular matrix lead to the long-term prolonged movement of the entire cell. This process is characterized by alternating phases of direct migration and changes of direction and polarization. The coordinated interaction of these phases suggests the existence of intermittency, strong space-time correlations, and a close relationship between units (cell-cell interaction). It is therefore an important question whether the long-term movement of the entire cell can still be understood as a simple diffusive behavior such as Brownian movement or a random walk or whether more advanced dynamic modeling concepts should be applied [10,11,12].

The change in shape and density of the cells during migration make it difficult to apply standard automatic single particle tracking (SPT) pipelines to extract the cell migration trajectory in phase contrast images acquired in time-lapse. These difficulties are even greater when collective motion is considered and a dense agglomerate of cells is present. To overcome such limitations, here, we propose a pipeline for segmentation and SPT extraction in phase contrast images of the wound healing scratch assay. The pipeline is original and follows the principle of Occam’s razor, based on a simple measure as linear binary patterns (LBPs), which results in being sufficient to classify the texture as cells or background by using a principal component analysis (PCA) and Gaussian mixture classification, the code is available at the git-hub repository https://github.com/riccardoscheda/AnomalousDiffusion (accessed on 1 November 2020). We chose the manual segmentation performed over one experiment as the ground truth. We further compared the performance of our pipeline with segmentation by Otsu thresholding [13] without manual adjustment of the parameters for different frames of the same image. For all the cases, the wound edge is approximated to a unique membrane and its dynamics approximated by the stochastic motion of a point on the membrane, i.e., a pseudo-particle. This choice is motivated by the fact that in the experiment under study, faster cells do not separate from the borders during wound closure. Therefore, the dynamics and the heterogeneity of the process are characterized through the collection of such SPT trajectories.

The paper is organized as follows: in the the Methods Section, we present step by step our pipeline for phase contrast image processing and SPT; in the Results Section, we show the trends of the pseudo-particle trajectories’ statistics for a wound healing scratch assay, performed with glioblastoma T98G cells; in the Conclusions Section, we discuss the performance and the SPT statistics of our pipeline, in comparison with the corresponding measurements obtained through the professional tool ImageJ [14].

## 2. Methods

### 2.1. Data

Glioma cells (T98G), derived from brain human tumor glioblastoma multiforme (GBM), were plated at a density of $1\times 10^{5}$ cells/cm${}^{2}$ on 35 (∅) mm sterile Petri dishes with a 10 (∅) mm glass microwell (MatTek Corporation, Ashland, MA, USA) suitable for optical microscopy. The cell culture, with a population doubling time (PDT) approximately of 28 h, as reported by the ATTC Company, which provided the cell line, was maintained in Gibco${}^{\mathrm{TM}}$ Minimum Essential Medium (MEM) with Earle’s salts (Fisher Scientific, Milano, Italy) supplemented with 10% fetal bovine serum, 1% L-glutamine, 1% sodium pyruvate, and antibiotics (1% penicillin and 1% streptomycin) inside the incubator at 5% of CO${}_{2}$ and 37 ${}^{\circ }$C. All chemicals were purchased from Merck KGaA (Darmstadt, Germany). After 48 h from seeding, the population covered the entire surface as a monolayer of confluent and tightly contacting cells. Using a sterile pipette tip for Gilson (10–200 μL), a scratch ranging 200–400 μm along the middle axis was done. Right after, the specimen was placed into the pre-heated microscope stage incubator in the motorized table of the inverted optical microscope Eclipse Ti (Nikon, Bologna, Italy). The phase-contrast micrographs of multiple visual fields, pre-selected along the narrow scrape by the NIS Elements AR 4.0 (Nikon, Bologna, Italy) software, were acquired at 100× magnification for 20 h at the rate of 4 frames/hour. The setup allowed the acquisition of time-lapse images of living cultured cells maintained in standard conditions for the entire duration of the experiment.

### 2.2. Image Processing

The aim of the pipeline was to identify the wound edges, which correspond to the free edge of the two cell layers, separated by the wound. The procedure was done in the following steps, which should be applied to all the frames of an experiment: (i) equalization, to make all the frames comparable; (ii) binarization, to separate the background regions from the cell layers; (iii) wound edge identification; and (iv) storage of the coordinates. We describe here two alternative procedures of binarization, the first based on texture classification and the second on hand drawing the wound edges over the images by using the professional tool ImageJ [14].

#### 2.2.1. Equalization of the Frames

To improve the difference of the wound borders from the background regions, we applied to all the frames contrast limited adaptive histogram equalization (CLAHE) [15].

**Figure 1:**
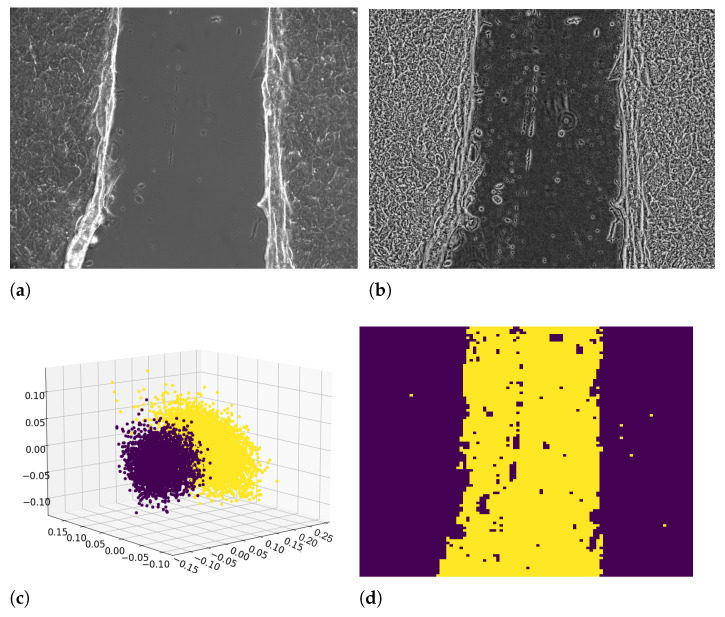
Original image (**a**); transformed image by using adaptive histogram equalization (**b**); 3D scatter plot of the first 3 principal components of the linear binary pattern (LBP) score PCA (**c**); the data points in the scatter plot are clustered by the Gaussian mixture model clustering algorithm; color labels refer to cells (blue) or background (yellow); binarized frame image by using texture analysis (**d**).

The image was divided into small blocks (tiles), with a tile size of 50×50, to enhance the difference between the cell border and the background (tile size is 8×8 by default in [16]). Then, each of these blocks was histogram equalized. Therefore, in a small area, the histogram would be confined to a small region (unless there was noise). If noise was there, it would be amplified. To avoid this, contrast limiting was applied. If any histogram bin was above the specified contrast limit (by default, 40 in [16]), those pixels were clipped and distributed uniformly to other bins before applying histogram equalization. This procedure increased the image contrast and enhanced the texture patterns (e.g., Figure 1b) by equalizing pixels’ intensity distribution of all the frames to a fixed range wider than the original ones.

#### 2.2.2. Image Binarization by Texture Analysis

Image binarization was performed by dividing each frame into 10,000 subimages (12×16 pixel subimages in 1200×1600 pixel image) and by classifying each of them as the background or cell layer on the bases of a score. The score was built to characterize the texture of each subimage and corresponded to the distribution of the local binary pattern (LBP) values for all the pixels of the subimage (scikit-image Python library [17], skimage.feature.local_binary_pattern). We considered grayscale images; thus, the LBP of each pixel corresponded to a scalar value. We calculate the LBP for a pixel by comparing the pixel with its 8 first neighbors. To each couple was assigned a score: if the central pixel value was greater than or equal to the neighbor pixel value, we assigned 1, otherwise, if the central pixel value was less than the neighbor pixel value, the score was 0. The LBP value of the pixel corresponded to the sum of these scores, ranging from 0 to 8, and contained information about the 3 × 3 square of pixels. The frequency of such LBP scores for each subimage was an array of 9 values representing a texture feature of the subimage. Therefore, each frame (image) was characterized by a matrix of 10,000 (sub-images) × 9 (LBP score) values. Principal component analysis (PCA) [18] was performed over the 9 dimensions of the texture score to separate the 10,000 subimages into two clusters: one corresponding to the background regions and one containing the cell layer regions (Figure 1c). Taking the first 5 principal components, the points belonging to the two clusters were classified and labeled (0 or 1) using the Gaussian mixture model clustering algorithm (scikit-learn Python library [19], sklearn.mixture.GaussianMixture). Each point in Figure 1c corresponds to a subimage; hence, the obtained binary color labels (yellow or blue) were used as binarized intensities for the corresponding subimages to build the binarized image (Figure 1d).

The performance of the algorithm with respect to the size of the subimages was studied in terms of the Pearson correlation of the segmented fronts with the ground truth for squared and rectangular shapes of different sizes. For complete tessellation of the image, it supported the choice of the subimages’ size of 12×16 pixels (see the Supplementary Material).

#### 2.2.3. Wound Edge Recognition from Binarized Images

**Figure 2:**
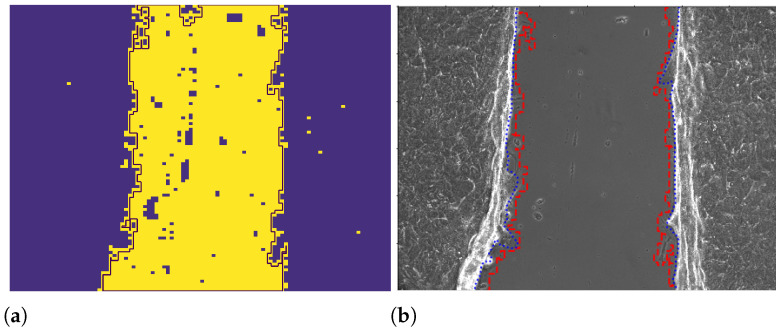
Image binarized by texture analysis with borders recognized by OpenCV-Python (**a**); example comparison of the borders obtained through the professional tool ImageJ (blue line) and the texture analysis (red dashed line) method superposed on the original image frame (**b**).

Contour lines can be easily recognized in a binarized image as the contour of 0 or 1 regions. We applied a function for contour identification, returning a list of all the contours in an image (OpenCV-Python library, findContours). In the frames, the longest contour line refers to the central part of the image until the two cellular fronts remain separate, identifying at the same time the background regions an the two borders of the cell layers (Figure 2a).

#### 2.2.4. Wound Edge Recognition with the ImageJ Professional Tool

The extraction of the fronts with the professional tool ImageJ for image analysis was performed by hand drawing the line of the front over each image (Figure 2b) and then by saving the corresponding coordinates for each frame and for left (L) and right (R) front in a .txt file [14].

#### 2.2.5. Wound Edge Recognition with Otsu Thresholding

For the simple thresholding of the images, we performed an adaptive histogram equalization (CLAHE) to improve the difference of the wound borders from the background, then we blurred the image with OpenCV Gaussian Blur, in order to have better results for the Otsu thresholding. Then, we applied Otsu thresholding on the image [17]. Then, we applied morphological transformations in order to have a smoother border of the wound. After morphological transformations, we collected the coordinates of the borders (OpenCV-Python library, findContours).

### 2.3. Pseudo-Particles’ Trajectories

The wound edge (L and R) of the cell layers was considered as a single homogeneous elastic membrane. The movement of such a membrane was tracked by means of *N* points uniformly distributed along its length as pearls on an elastic necklace. To derive the coordinates of the *N* pseudo-particles, we interpolated the wound edges’ 2D coordinates as a function of the front length (scipy Python library, scipy.interpolate.interp1d), and then, we computed the coordinates of the *N* pseudo-particles uniformly distributed along its length [20].

The collection of the *N* points constituted the collection of pseudo-particles, and for each of them, an SPT was built by considering its coordinates in the time sequence of the experiment frames. The SPTs in 2D were allowed to cross because of invaginations and protrusions of the front, despite the cells being attached to each other and the membrane of the wound edge being considered as a unicum. However, the average displacement along the membrane was approximately zero because it was constrained by the geometry of the system and the microscope field.

The pseudo-particle *n* at time $t_{k}$ was defined by the coordinates of the *n*-th pseudo-particle in the *k*-th frame of the image. The collection of the coordinates of the pseudo-particle *n* for all the frames represented the trajectory of the pseudo-particle *n*. Thus, the dynamics of the membrane could then be tracked by working on a matrix N×M, where *N* is the number of tracked pseudo-particle and *M* is the number of frames. The latter represent the time steps of the sampling.

### 2.4. SPT Statistics

To study the SPT statistics, we applied the discrete version of mean squared displacement (MSD) and autocorrelation functions (ACFs). In fact, discrete SPT statistics can be performed directly on an SPT *N* (pseudo-particles)×M (frames) matrix dataset, one for the *x* coordinate and one for the *y* coordinate, where the frame index k=0,1,2…,M−1 corresponds to the sampling time, i.e., the time step of the process, and n=1,2…,N corresponds to the index of the pseudo-particle. Statistics on the single trajectory could be performed as matrix operations along the *M* columns, while the ensemble average could be performed by averaging over the *N* rows of the transformed matrix. In the present work, we considered only the movement toward the free edge of the layers for the sake of simplicity, i.e., the *x* component of the quantities of interest. For statistical analysis, we applied a shift to the pseudo-particle position such that 〈X(t=0)〉=0. Moreover, due to the lack of long stationary trajectories, we considered here only ensemble averages:

(1) $$E\left(Y\left(k\right)\right)=\frac{1}{N}\sum_{n=1}^{N}y_{n}\left(k\right)\;,$$

where $y_{n}\left(k\right)$ is the value of the variable *Y* for the *n*-th pseudo-particle at time t=k. The velocity of the pseudo-particle is defined as the increment of the pseudo-particle position *X* per unit sampling time (0.25 h):

(2) V(τ)=X(τ+1)−X(τ),τ=0,1,2…,M−2.

The increments of the velocity per unit sampling time are defined as the following:

(3) A(τ)=V(τ+1)−V(τ),τ=0,1,2…,M−3.

The autocorrelation function $ACF_{Y}$ for the generic variable *Y* reads:

(4) $$ACF_{Y}\left(\tau \right)=\frac{E[\left(Y\left(t_{0}\right)-\mu_{t_{0}}\right)\left(Y\left(t_{0}+\tau \right)-\mu_{t_{0}+\tau }\right)]}{\sigma_{t_{0}}\sigma_{t_{0}+\tau }}\;,\;\tau =0,1,2\ldots ,M-1\;,$$

where the initial time is $t_{0}=0$ and $\sigma_{k}$ and $\mu_{k}$ represent respectively the standard deviation and the mean of the variable *Y* at time t=k.

The mean squared value (MSY) for the generic variable *Y* reads:

(5) $$MSY\left(\tau \right)=E[{\left(Y\left(t_{0}+\tau \right)-Y\left(t_{0}\right)\right)}^{2}]\;,\;\tau =0,1,2,\ldots ,M-1\;,$$

where the initial time is again $t_{0}=0$.

### 2.5. Fit Procedure

All the fits were performed through a ordinary least squares (OLS) regression (scipy Python library, scipy.optimize.curve_fit), which returned the optimized parameters of the model and their matrix of covariance [20]. Poissonian uncertainty for counts in histograms was considered. We further compared (results not shown) the parameters estimated by OLS with the ones obtained by the maximum likelihood estimate (MLE) (stats Python library, scipy.stats.rv_continuous.fit).

## 3. Results

We considered a single field in an experiment of the wound healing scratch assay (without external stimuli applied to the cell substrate) as the test image.

**Figure 3:**
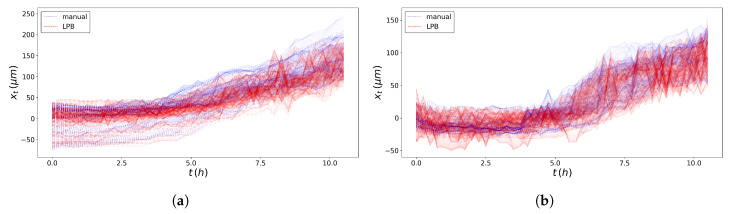
Comparison of the single particle trajectories obtained with the professional tool ImageJ (blue line) and the texture analysis (red or dashed line) method for the left wound edge (**a**) and the right wound edge (**b**), for $N=10^{3}$.

The trends of the *N* SPTs obtained through the texture analysis for the experiment under study are compared with the ones obtained by using the professional tool ImageJ in Figure 3. SPTs from the right front are also mirrored.

**Figure 4:**
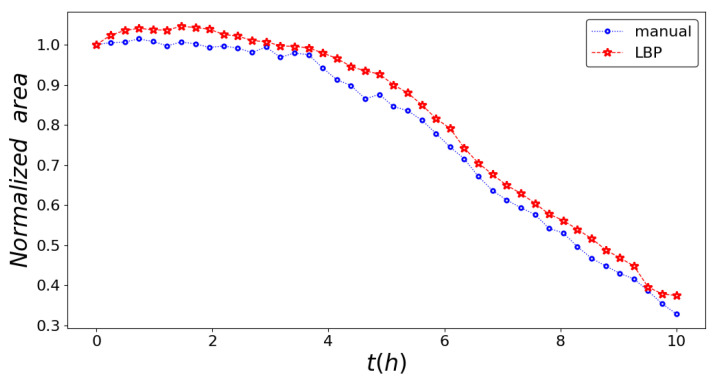
Normalized area between the wound edges during wound closure as a function of time for the texture analysis (blue line) and the professional tool ImageJ (red line).

In Figure 4, we display the temporal trends of the area between the wound edges during wound closure, estimated by using the texture analysis in comparison with the wound edges recognized manually.

**Figure 5:**
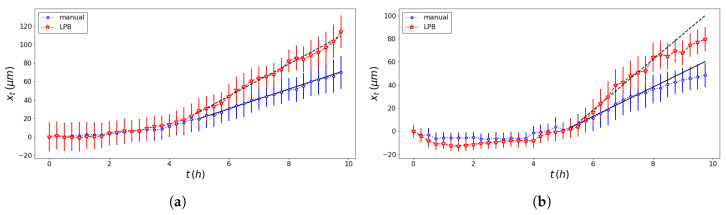
Comparison of the average position of the pseudo-particle obtained with the professional tool ImageJ (blue dotted line) and the texture analysis (red dashed line) and their linear OLS best fit (black dashed line; see Table 1 for details) for the left wound edge (**a**) and the right wound edge (**b**).

**Figure 6:**
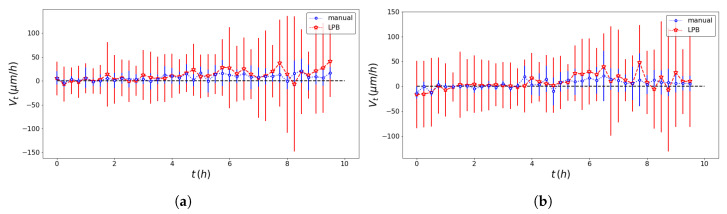
Comparison of the ensemble averaged pseudo-particle velocity obtained with the professional tool ImageJ (blue dotted line) and the texture analysis (red dashed line) for the left wound edge (**a**) and the right wound edge (**b**).

**Figure 7:**
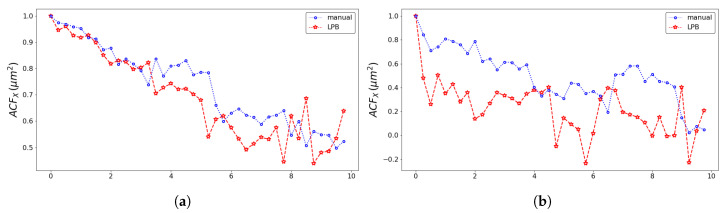
Comparison of the coordinate *X* autocorrelation function obtained with the professional tool ImageJ (blue dotted line) and the texture analysis (red dashed line) for the left wound edge (**a**) and the right wound edge (**b**).

**Figure 8:**
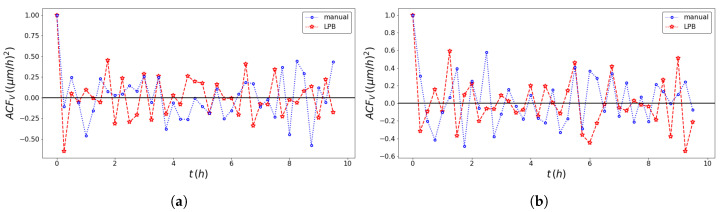
Comparison of the velocity autocorrelation function obtained with the professional tool ImageJ (blue dotted line) and the texture analysis (red dashed line) method for the left wound edge (**a**) and the right wound edge (**b**).

**Figure 9:**
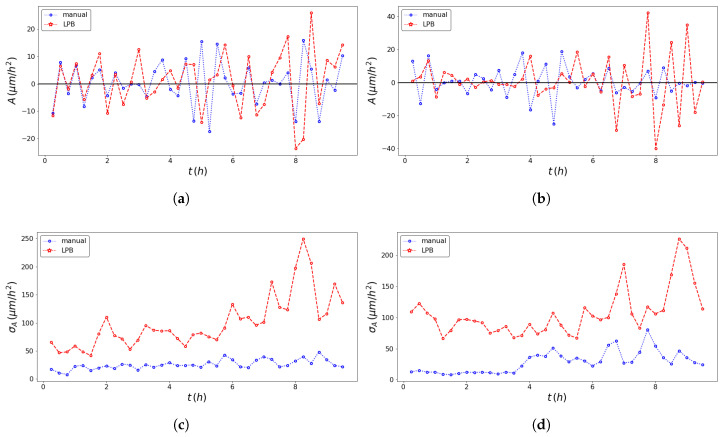
Comparison of the ensemble averaged pseudo-particle acceleration trajectory obtained with the professional tool ImageJ (blue dotted line) and the texture analysis (red dashed line) method for the left wound edge (**a**) and the right wound edge (**b**); comparison of the standard deviation of pseudo-particle acceleration of the ensemble of pseudo-particles obtained with the professional tool ImageJ (blue dotted line) and the texture analysis (red dashed line) method for the left wound edge (**c**) and the right wound edge (**d**).

**Figure 10:**
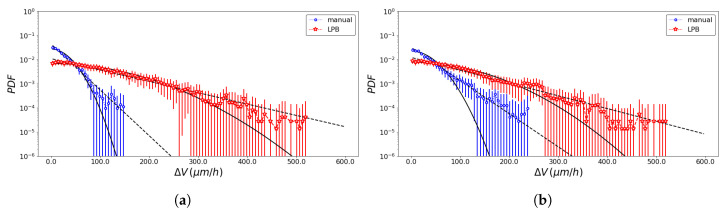
Comparison of the velocity increments (absolute value) obtained with the professional tool ImageJ (blue) and the texture analysis (red) method for the left wound edge (**a**) and the right wound edge (**b**); the best OLS fit of the frequencies of a exponential (dashed line) and a normal distribution (bold line) is shown for the two methods (see Table 2 for details).

The pseudo-particle average position and average velocity for the two methods of analysis are displayed in Figure 5 and Figure 6. The ACFs of the pseudo-particle position and velocity along the *x* coordinate are compared for the two methods of analysis in Figure 7 and Figure 8, respectively. A regime with stationary increments of the velocity was identified for the time range between 5 h and 8 h (Table 1), corresponding to the duration of the regime with constant drift velocity in the ensemble averaged position (Figure 5). The medium could be roughly approximated as viscous, and a constant velocity implies constant force, on average, applied against friction by the cells. This stationary regime with constant drift velocity was supported by zero correlation in the VACF(Figure 8) and by the symmetric distribution of velocity increments with a zero average. For such a time range, the distribution of the instant acceleration (velocity increments) along the *x* coordinate is shown in Figure 9 for the two methods of image segmentation. The tails of these distribution are compatible with both the exponential and the Gaussian scaling, with comparable characteristic scales (Table 2). However, the linear decay of the tails in Figure 10 suggests that a truncated-exponential decay is more plausible. To estimate the consistency between the two methods, we computed the Pearson’s correlation (Table 3) for their estimates of the pseudo-particles’ coordinates, i.e., the entire collection of estimated position for the *x* and *y* coordinates, the average position 〈X〉, the average velocity 〈V〉, and the average velocity increments 〈A〉 for $N=10^{3}$ and M=40.

## 4. Conclusions

We present an original method to extract a 2D discrete representation of the wound edge in phase contrast images acquired in time-lapse by texture analysis, and we compare the results with the ones obtained by using the professional tool ImageJ and by Otsu thresholding (see the Supplementary Material for edges derived by thresholding). The dynamics of the wound edges is defined by the SPT of *N* pseudo-particles uniformly distributed along the length of the fronts.

Thus, discrete SPT statistics can be performed directly on the SPT *N* (pseudo-particles) ×M (frames) matrix dataset for the *x* coordinate (crossing the wound gap).

We compare SPT statistics of the data obtained by hand drawing with the texture analysis: average values, squared mean values, and the autocorrelation function of position and velocity. The two approaches lead to consistent results in terms of the trends of the dynamics (qualitative analysis) and in terms of Pearson’s correlation (Table 3). By a visual check, the texture analysis appears more capable of recognizing lamellipodium protrusions than the professional tool ImageJ, because such tiny structures could be occasionally missed by human recognition (Figure 2). On the other side, the automatized procedure may also produce artifacts in the front profile, for example it would consider as part of the cell layer pieces of dead cells remaining in the middle of the wound when the cells at the wound edges get close to them. For these reasons, the wound edges detected by texture analysis are associated with larger fluctuations between different frames than the ones detected manually. For the same reasons, the pseudo-particles position fluctuates more in the texture analysis dataset between different frames, generating larger tails in the distribution of increments along the *x* coordinate for the velocity (and position) of the pseudo-particle (Figure 10) and larger mean squared velocity, in comparison to the one obtained by the professional tool ImageJ method. Despite such discrepancy, the average drift velocities (Figure 5), which correspond to the mean of the distribution of the position increments and the average velocity increments (Figure 10), are comparable.

Finally, by studying the SPT statistics, we are able to identify an intermediate regime characterized by a constant average of the cellular front velocity and by exponential tails for the velocity increments’ distribution (Table 2). We leave the full characterization of the stochastic process and the biological meaning, which are beyond the scope of the present paper, to future research with an enlarged cohort of experiments, in order to increase the statistics, but also to characterize the inherent variability of the phenomena.

## Figures and Tables

**Table 1:** Estimated average drift velocity of the cell front $v_{d}$ and the lag time of quiescence $\tau_{1}$ with their corresponding standard error obtained through OLS regression of the model and goodness of fit (Adj. R-squared). The second time scale $\tau_{2}=8$ h is estimated from Figure 5b.

Model	Front	Method	$v_{d}\;\pm$ SD (μm/h)	$\tau_{1}\;\pm$ SD (h)	Adj. R-Squared
${x\left(t\right)|}_{\tau_{1}<t<\tau_{2}}=v_{d}\cdot \left(t-\tau_{1}\right)$	L	ImageJ	19.5±0.3	4.49±0.03	0.990
	L	texture analysis	17.4±0.3	4.75±0.03	0.984
	R	ImageJ	23.9±0.4	5.25±0.03	0.987
	R	texture analysis	21.8±0.5	5.25±0.03	0.975

**Table 2:** Estimated parameters with their corresponding standard error obtained through OLS regression of the corresponding model and goodness of fit (Adj. R-squared).

Model	Front	Method	λ± SD (μm)	σ± SD (μm${}^{2}$)	Adj. R-Squared
$P\left(|\Delta V|\right)=2\cdot G(|\Delta V|;0,\sigma^{2})$	L	ImageJ		29±1	0.982
	L	texture analysis		117±2	0.980
	R	ImageJ		36±1	0.976
	R	texture analysis		103±2	0.982
$P\left(|\Delta V|\right)=\frac{1}{\lambda }e^{-|\Delta V|/\lambda }$	L	ImageJ	23±1		0.976
	L	texture analysis	92±2		0.948
	R	ImageJ	31±1		0.987
	R	texture analysis	82±2		0.949

**Table 3:** Pearson correlation of the collection of the pseudo-particles’ coordinates, *x* and *y*, the average position 〈X〉, the average velocity 〈V〉, and the average velocity increments 〈A〉 estimated by ImageJ with the one estimated by texture analysis for $N=10^{3}$.

Front	Variable	Pearson’s Coeff.	*p*-Value
L	*x*-coords	0.942	0.0
	*y*-coords	0.985	0.0
	〈X〉	0.997	1 × 10${}^{-50}$
	〈V〉	0.547	1 × 10${}^{-4}$
	〈A〉	0.275	0.08
R	*x*-coords	0.913	0.0
	*y*-coords	0.986	0.0
	〈X〉	0.997	1 × 10${}^{-48}$
	〈V〉	0.630	1 × 10${}^{-6}$
	〈A〉	0.156	0.19

## Data Availability

Not applicable.

## Supplementary Materials

The following are available online at https://www.mdpi.com/1099-4300/23/3/284/s1.
